# Eastes v. Russ: An Interesting Case

**Published:** 1913-06-07

**Authors:** 


### EASTES v. RUSS.

### An Interesting Case.

As we announced last week, this Chancery case resulted in judgment for the defendant, with costs. The issues at stake are of some little importance, and Mr. Justice Sargant's summing-up of them sets forth the salient facts with great lucidity. Briefly, both plaintiff and defendant are qualified specialists in bacteriology and pathology. The former was one of the first to start a private laboratory for work of this nature in London, and he has made a conspicuous success of the undertaking. Several hospitals, indeed, where no definitely appointed pathologist does the work have found it convenient to have tissues reported on and similar work done for them by Dr. Eastes, whose business has grown on such a scale that he employs a quite considerable staff of qualified and unqualified assistants. Those who thus accept employment are required, as a preliminary, to accept certain conditions, one of which (number 12) seeks to prohibit the employee from engaging in any similar work, on his own account or for any rival institution, within ten miles of Queen Anne Street, W., where Dr. Eastes' Laboratories were. After leaving the plaintiff's employment, the defendant set up a pathological laboratory of his own quite a short distance away; and hence the action brought by his late employer to restrain him from continuing thus to practise.

Curiously enough, Dr. Russ appears never to have signed this form; but he did not rely on that as a defence, and his advisers raised three other defences. The first of these was that the proper construction of clause 12 merely prevented him from doing other work during his actual engagement with the plaintiff: in other words, it was intended merely to secure that he should give his whole attention to the work for which he was being paid.

The second point raised by the defence was that, even if the foregoing construction was held to be wrong, the terms used were so ambiguous that the defendant was taking a quite reasonable view in thus construing it, and should therefore not be penalised by the enforcement of a condition to which he had never intended to consent. The third point was that the condition should be void and unenforceable, as being more stringent and far-reaching than was necessary for the proper protection of the plaintiff.

To begin with, the Judge pronounced against the defence upon this last point. He admitted that the prohibition seemed to extend for the whole of the defendant's life, since no time limit was mentioned; but he did not feel disposed to hold the whole clause void as unreasonable. He did, however, find that the all-important clause 12 should legally be binding only during the duration of the engagement; and for this reason he gave judgment in favour of the defendant. He further stated that he did consider the clause so ambiguous that the defendant would be justified in putting the construction upon it which he (Mr. Justice Sargant) himself did: so that practically he upheld both the first and second lines of the defence, but not the third one. He also agreed that the handicap inflicted on Dr. Russ by Dr. Eastes' construction of the document would be monstrous, although recognising that the latter is put to some hardship by the removal of the restriction altogether, entailed by the judgment.

In these opinions his Lordship will probably be supported by most of those who read his very admirable presentation of the facts of the case and the application to them of the principles of law. There can be no doubt that Dr. Eastes fully intended to make it a condition that no employee should set up in opposition within ten miles. Such a condition is very drastic, but if men can be found willing to subscribe to it, so much the better for Dr. Eastes.

